# Supplementary material for: TP53 Status, Patient Sex, and the Immune Response as Determinants of Lung Cancer Patient Survival
Source: Cancers (Basel). 2020 Jun 11;12(6):1535. doi: 10.3390/cancers12061535 (PMC7352604; doi:10.3390/cancers12061535)
Supplement: Supplementary file 1 [file cancers-12-01535-s001.zip › Supplementary figures.pdf]

Supplemental Materials: TP53 Status, Patient Sex, and the Immune Response as Determinants of Lung Cancer Patient Survival

Donald Freudenstein Cassandra Litchfield, Franco Caramia, Gavin Wright, Ben Solomon, David Ball, Simon P. Keam, Paul Neeson, Ygal Haupt and Sue Haupt

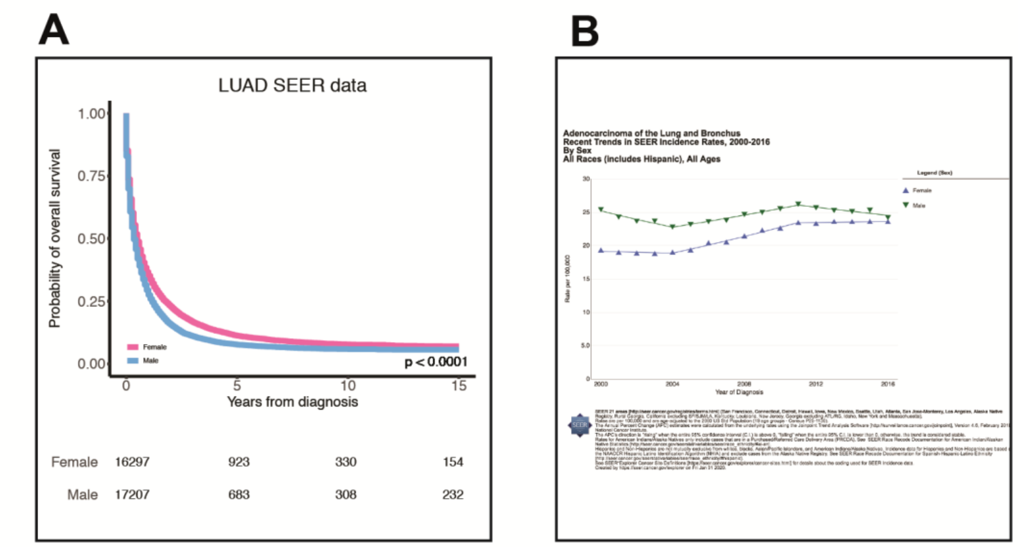

Figure 1. US LUAD incidence is lower in females and they outlive their male counterparts**.** US SEER population data analysed for **(A)** overall survival of female and male patients with LUAD (females: pink and males: blue); and (**B**) relative incidence according to patient sex (females: blue; males: green), with last data collected in 2016. Significance is indicated the *p*-value for the log-rank test of differences in survival time between females and males, with significance defined as < 0.05. See Methods section for description of methodology and data sets utilised in survival analysis.

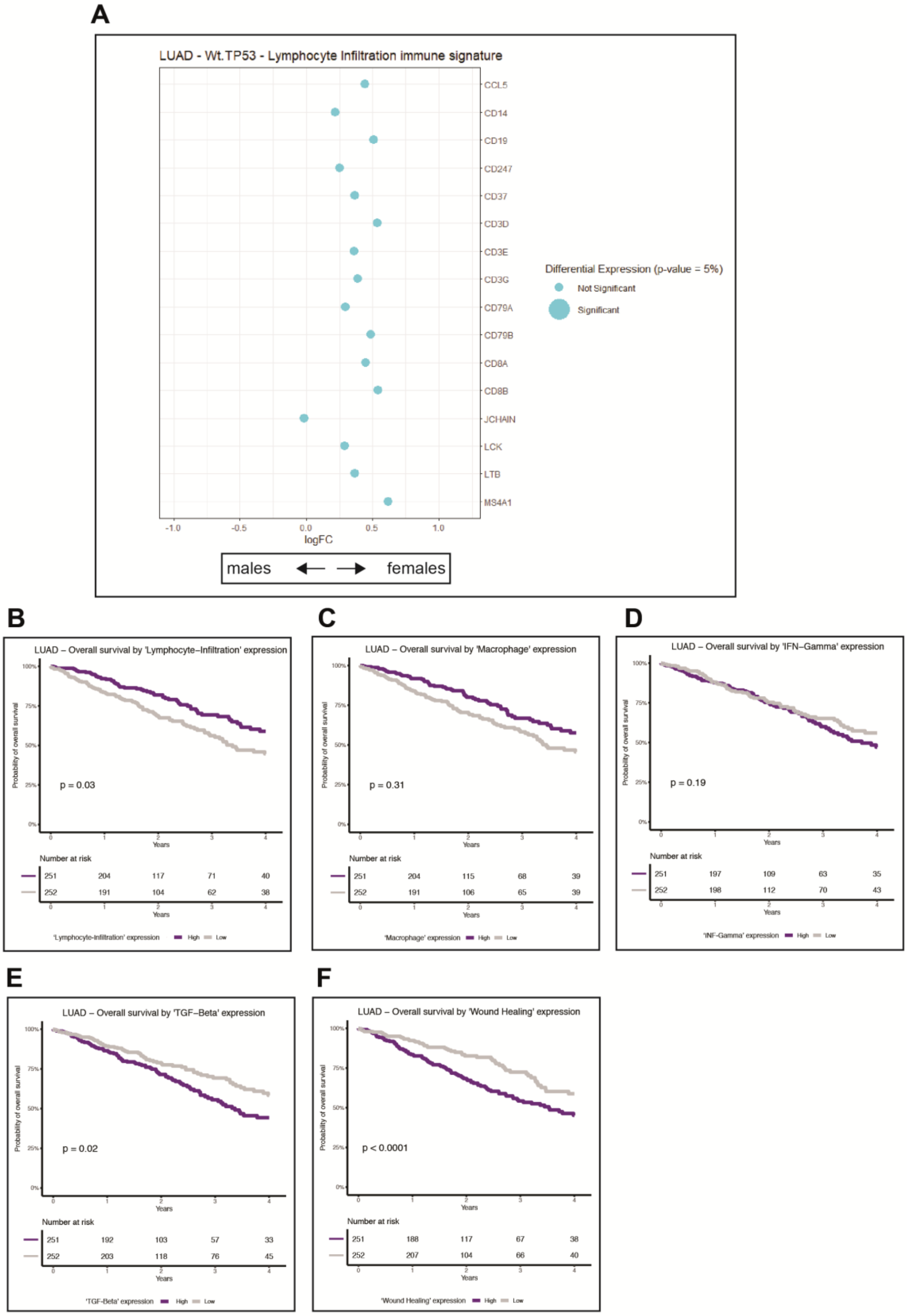

Figure 2. Immune signatures of LUAD have predictive value for survival. (**A**) GSEA comparison between males and females of the Lymphocyte infiltration immune gene signature of LUAD in TCGA data sets. Overall survival of LUAD patients corresponding to relative expression of immune signature genes for (**B**) Lymphocyte infiltrate expression; (**C**) Macrophage expression;(**D**) IFN-γ expression; (**E**) TGF-β expression; and (**F**) Wound healing expression. The numbers of patients corresponding to each cohort, stratified for high (purple) or low (grey) expression of the respective genes, are tabulated below each plot. *p* is the p-value for the log-rank test of differences in survival

time (significant p-value < 0.05). Significance is indicated the p-value for the log-rank test of differences in survival time between females and males, with significance defined as <0.05. See Methods section for description of methodology and data sets utilised in survival analysis.

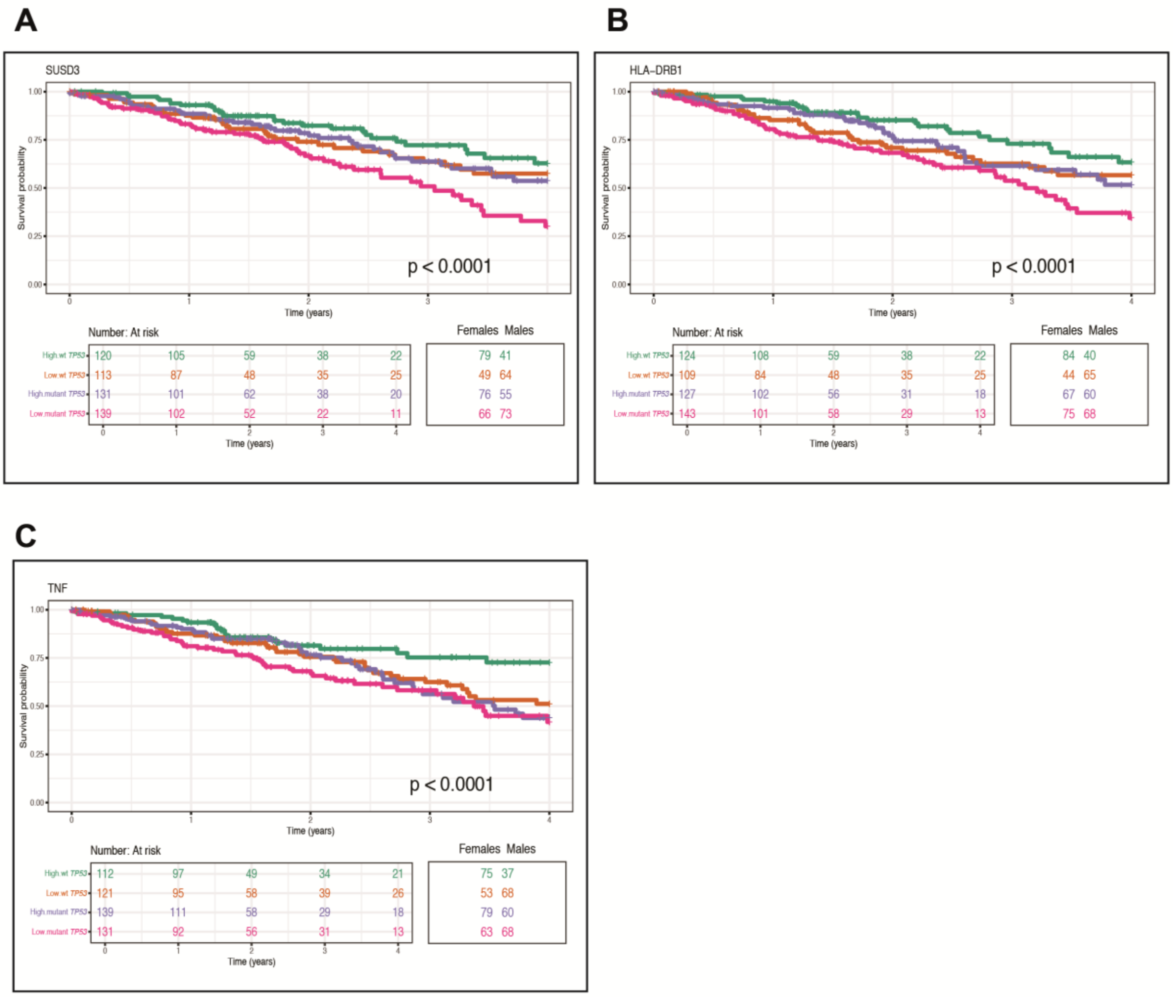

Figure 3. Relative expression of individual immune genes have predictive value for survival of LUAD patients. Overall survival of LUAD patients in TCGA corresponds with relative expression of (A) SUSD3; (B) HLA-DRB1; and (C) TNF. Significance is indicated the p-value for the log-rank test of differences in survival time between females and males, with significance defined as <0.05. The numbers of males and females corresponding to each cohort, stratified for high or low expression of the respective genes, in either a wt or mutant *TP53* context are tabulated below each plot. 'p' is the p-value for the log-rank test of differences in survival time (significant p-value < 0.05). See Methods section for description of methodology and data sets utilised in survival analysis.

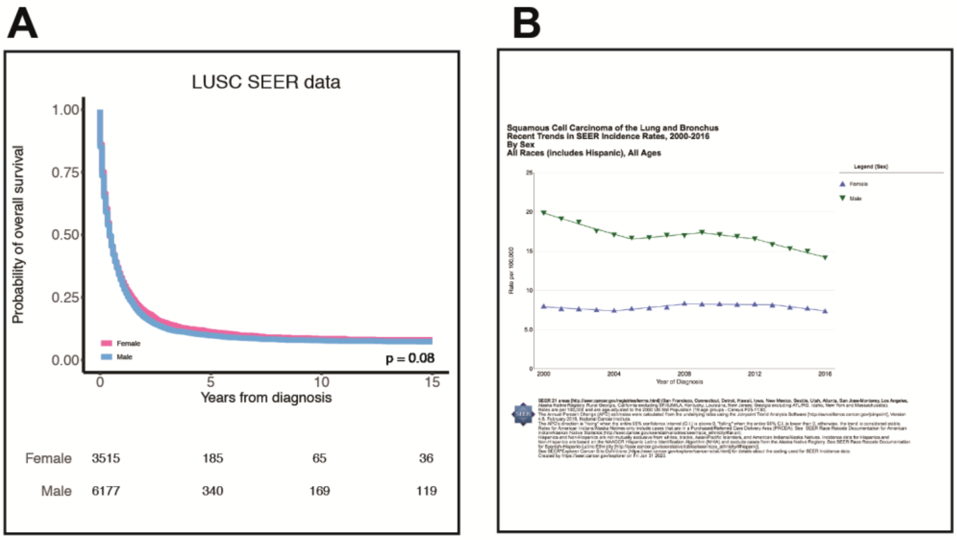

Figure 4. Overall LUSC patient survival is similar between US males and females, with higher incidence in males. US SEER population data analysed for (**A**) overall survival of female and male patients with LUSC (females: pink and males: blue); and (**B**) relative incidence according to patient sex (females: blue; males: green), with last data collected in 2016. Significance is indicated the *p*-value for the log-rank test of differences in survival time between females and males, with significance defined as <0.05. See Methods section for description of methodology and data sets utilized in survival analysis.

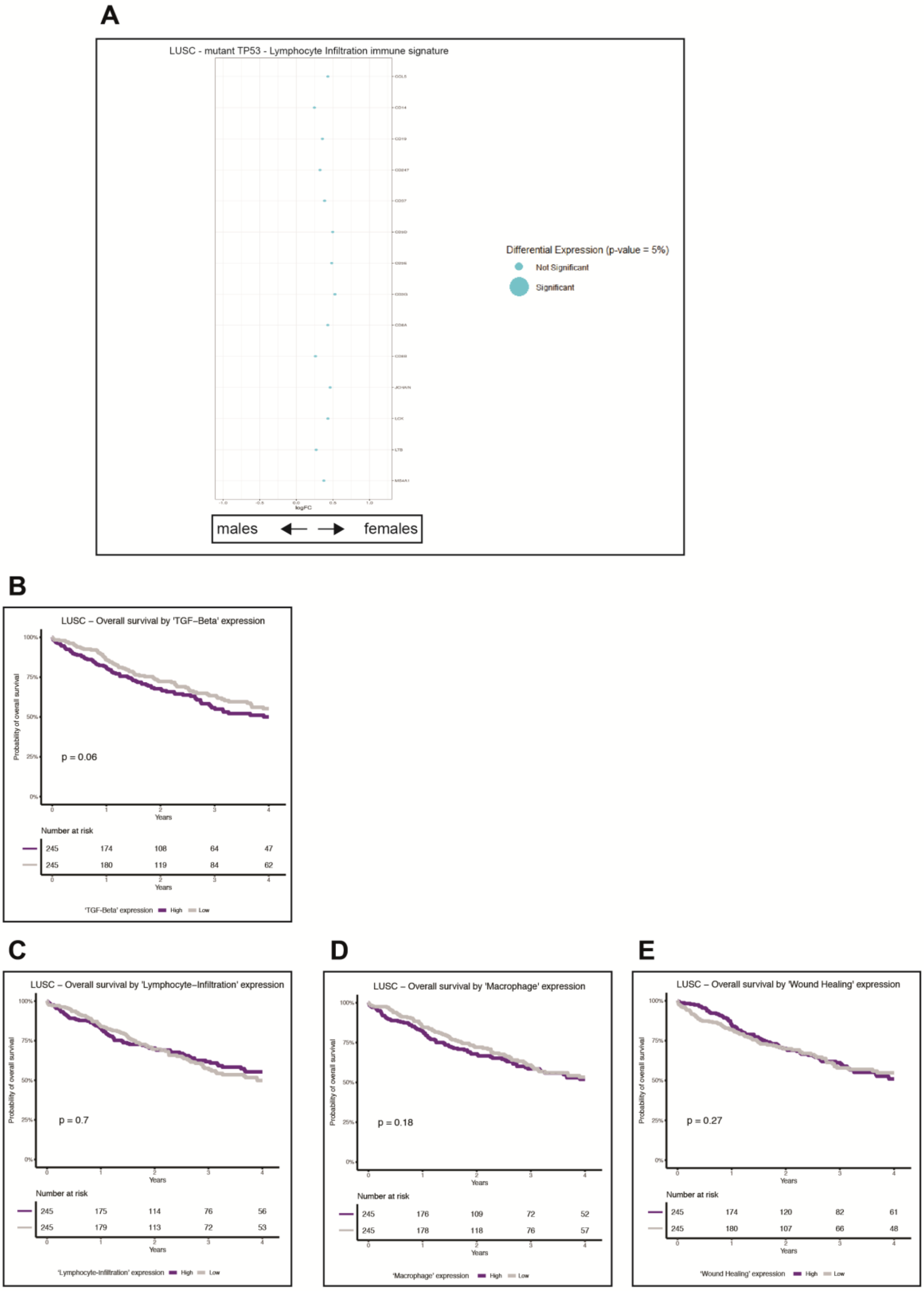

Figure 5. Immune signatures among LUSC patients are not predictive of survival. (**A**) GSEA comparison of Lymphocyte infiltration immune gene signature among male and female LUSC with mutant *TP53*, in TCGA data sets. Overall survival of LUSC patients corresponding to relative expression of immune signature genes for (**B**) TGF-β expression; (**C**) Lymphocyte infiltrate expression; (**D**) Macrophage expression; and (**E**) Wound healing expression. Significance is indicated the *p*-value for the log-rank test of differences in survival time between females and males, with significance defined as <0.05. The numbers of patients corresponding to each cohort, stratified for high (purple) or low (grey) expression of the respective genes, are tabulated below each plot. '*p*' is the

p-value for the log-rank test of differences in survival time (significant *p*-value < 0.05). See Methods section for description of methodology and data sets utilized in survival analysis.

© 2020 by the authors. Licensee MDPI, Basel, Switzerland. This article is an open access article distributed under the terms and conditions of the Creative Commons Attribution (CC BY) license (http://creativecommons.org/licenses/by/4.0/).
